# Micron-Scale Biogeography of Seawater Biofilm Colonies at Submersed Solid Substrata Affected by Organic Matter and Microbiome Transformation in the Baltic Sea

**DOI:** 10.3390/ma15186351

**Published:** 2022-09-13

**Authors:** Maciej Grzegorczyk, Stanislaw Pogorzelski, Paulina Janowicz, Katarzyna Boniewicz-Szmyt, Pawel Rochowski

**Affiliations:** 1 Institute of Experimental Physics, Faculty of Mathematics, Physics and Informatics, University of Gdańsk, Wita Stwosza 57, 80-308 Gdańsk, Poland; 2 MGE, Lipowa 7, 82-103 Stegna, Poland; 3 Department of Physics, Gdynia Maritime University, Morska 81-87, 81-225 Gdynia, Poland

**Keywords:** Baltic waters, submersed solid biofilm, confocal microscopy, biomorphology architecture, trophic state indexes, bioassessment indicators

## Abstract

The aim of this research was to determine temporal and spatial evolution of biofilm architecture formed at model solid substrata submersed in Baltic sea coastal waters in relation to organic matter transformation along a one-year period. Several materials (metals, glass, plastics) were deployed for a certain time, and the collected biofilm-covered samples were studied with a confocal microscopy technique using the advanced programs of image analysis. The geometric and structural biofilm characteristics: biovolume, coverage fraction, mean thickness, spatial heterogeneity, roughness, aggregation coefficient, etc., turned out to evolve in relation to organic matter transformation trends, trophic water status, microbiome evolution, and biofilm micro-colony transition from the heterotrophic community (mostly bacteria) to autotrophic (diatom-dominated) systems. The biofilm morphology parameters allowed the substratum roughness, surface wettability, chromatic organisms colony adaptation to substrata, and quorum sensing or cell to cell signaling effects to be quantitatively evaluated. In addition to the previous work, the structural biofilm parameters could become further novel trophic state indicators.

## 1. Introduction

In most aquatic ecosystems, a complex mixture of algae, cyanobacteria, heterotrophic microbes and detritus called periphyton can be found attached to submerged surfaces [1]. The biofilm development consists of subsequently appearing phases, starting from conditioning, reversible adsorptive film to finally complex mature macrofouling community structurally differentiated [2]. The spatial organization of complex natural biofilm colonies is critical to understanding the interaction of the individual taxa that comprise a community. The consortium ranges in size from a few tens to a few hundred microns in radius and is spatially differentiated. Bacteria are micron-sized, and many of the forces and factors that underlie their distribution patterns operate at micron scales and are qualitatively different from the large-scale factors, such as a trophic state status, primary production or man-made industrial pollution stress. These features include the host and inanimate surface architecture and roughness on which the microbes reside as well as local gradients of nutrients and biogenic elements. The microbes themselves are key components of the micro-scale environment, particularly in biofilms and other densely populated habitats, appearing as a substratum for attachment of further microbes, creating spatial structure, and acting as a point source for diffusible metabolites. Particular biofilm features such as: short generation time, sessile nature and fast responsiveness to environmental condition stressors make them an effective seawater chemistry monitoring tool, as demonstrated in recent Baltic Sea eutrophication studies [3]. In this research, geometric and structural, commonly evaluated biofilm morphology characteristics such as: biovolume, coverage area fraction, area to volume ratio, spatial heterogeneity, number of species, mean thickness, roughness, fractal dimension in 2D, Hopkin’s aggregation index, aggregation coefficient (AC) etc. were on-line determined from confocal reflection microscopy (COCRM) images data [4], processed with the advanced graphical programs (CMEIAS, COMSTAT, ImageJ, PHLIP etc.) on samples collected at Baltic sea submerged artificial solid substrata. The aim of the study was threefold: establishment the time-dependent biofilm structure parameters evolution as a basis to create the modified biofilm kinetics model, to quantify their correlations to the water body trophic state indexes, to follow the seasonal evolution of an autotrophic system, and to derive a quantitative response of the biofilm consortium to the solid surface features (roughness architecture, substratum wettability and color).

## 2. Materials and Methods

Several artificial solid materials: glass, metallic (Al, brass alloy, Cu, Fe), polymeric (PVC, PMMA, PC, PTFE), rubber, etc., of varying surface energy (and wettability energetics) were submerged at a depth of 0.5 m in near-shore waters of the southern Baltic Sea as biofilm collectors, for a certain time, as described elsewhere [3]. Biofilm accumulation time ranged from 1 to 24 days; probes were studied every month from April to November 2018. No mechanical surface treatment procedure was applied to the test surfaces apart from the ultrasonic cleaning process by means of acetone and ethanol. Each test surface was used as received; the surface roughness of the samples R_rms_ ranged from 2.3 to 5.6 μm. The subsequent stages of biofilm creation (i.e., the characteristic time of the mature film creation) were specified from the surface coverage vs. time plot, as shown in Figure 8 of [3]. It allowed us to determine the optimal time of biofilm collection (over which no significant colony structural features evolution took place) at the particular seasonal year period. As a result, the optimal sample submersion period was attributed to the specific study place in relation to the spatial-seasonal organic matter evolution. Particularly, on 10 sampling days (T = 2, 4, 6, 8, 10, 12, 14, 16, 18, 20 days), 3–4 biofouled substrata were taken off with 2-day intervals.

Tested surfaces, to study the surface roughness effect on biofouling accumulation, were prepared by the abrasive polishing process. It allowed us to create mono-directional morphologically oriented roughened surfaces. The flat Al rectangular plates (of dimensions: 78 × 25 × 1.5 mm) were one side polished on a different grit (80–600) sandpapers to produce a wide range of surface roughness (R_rms_ = 0.22–19.6 μm, where R_rms_ is the root-mean-square value of the vertical roughness distribution).

In order to characterize the wettability of model solid sampler surfaces, the water contact angle (WCA) measurements were performed and the wettability energetics parameters, i.e., surface free energy (γ_SV_), contact angle hysteresis (CAH), 2D surface adsorbed film pressure, works of adhesion (W_A_) and spreading (W_S_) were determined, as described in detail elsewhere [5,6]. The trophic status was evaluated with the parameters: pH, dissolved O_2_, phosphate, nitrite, nitrate, ammonium concentrations using a spectrometric method. Primary production, Chlorophyll a *(Chl. a)*, nitrogen, phosphorus concentrations were taken from the SatBałtyk system database (available at http://satbaltyk.iopan.gda.pl). Biofilm wet mass (BWM) was chosen as a direct measure of the solid biofilm collection efficiency. A biofilm material, from the known area (a few cm^2^), was scratched using a spatula, and then weighted with a microbalance (Δm = 10^−5^ g).

A confocal scanning microscopy system working under the reflection mode configuration was used for biofilm surface morphology analyses, as described in [6]. To derive surface roughness (SR) profiles of the sample, a confocal microscopy system (Axiovert 200M, Carl Zeiss, Jena, Germany), and contour GT optical profilometer (Bruker, Ettlingen, Germany) were used.

From a vertically-separated stack of sample images, detailed 3D projections of the surface morphology, surface geometric and structural signatures were evaluated by means of the advanced graphical analysis programs (CMEIAS 1.2, COMSTAT 2, ImageJ 1.49 v, Helicon Focus 8.1.1, PHLIP). In brief, the image thresholding after data binarization was applied, and the biofilm parameters are calculated from the image stacks [7].

In particular, the following 3D biofilm structure parameters were derived, with the corresponding references pointed to where the mathematical routines of their evaluation can be found:

- biovolume (V) is the number of foreground pixels, N, in an image stack multiplied by the voxel volume [8];
- coverage area fraction (f); the fraction of pixels comprised by a biofilm material for each image cross section is calculated by PHLIP [7], thus the fraction f is derived as the ratio of foreground pixels to the total number of pixels for a given cross-section;
- area to volume ratio; its final value is determined by calculating the ratio A/V, where the surface area (A) of the said image stack represents the number of foreground pixels, which are attached to one neighboring background pixel [9];
- mean thickness and roughness; are these parameters, which are often chosen to describe the biofilms topography. The average value of the resulting pixel height h distribution, for every point located in the x-y surface plane, stands for the mean thickness (M) [7]. Surface roughness fluctuation dimensionless coefficient (η) defined as: η = SD/R_rms_ (SD represents the standard deviation from the mean) was introduced to standardize the surface undulation degree [6]. Consequently, the roughness fluctuation coefficient (η) is obtained by dividing the standard deviation of the distribution by M [9];
- fractal dimension in a 2D plane: the fractal dimension parameter is a measure of the biofilm boundary roughness complexity (development degree) between foreground and background pixels in a cross-section at height z, with values varying between 1 and 2. Higher values of the fractal dimension parameter point to a wavier (roughened) biofilm boundary. The fractal dimension parameter is calculated, as described in [10];
- Hopkin’s aggregation index reflects the biofilm colony dispersion state and takes values <2, for randomly distributed biofilm species [11];
- aggregation coefficient (AC) to quantify the aggregation levels in a biofilm culture using 3D micrographs [12], reflecting the changes in average species aggregate dimensions and proportions.

Spearman’s rank correlation routine was adopted to establish cross-correlations between the geometric-structural biofilm parameters and trophic state indexes.

## 3. Results and Discussion

### 3.1. Biofilm Morphology

**Figure 1:**
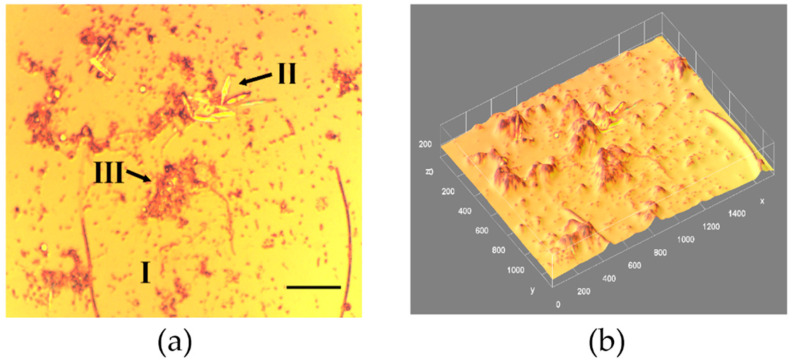
(**a**) A microscopic projection image of a marine biofilm grown on glass slides (14-day old) in Baltic Sea coastal waters (Gulf of Gdańsk, Poland) collected in August 2018; covered area 460 × 570 μm. Black arrows indicate the different reflective structures containing bare glass substratum (I), silica frustules of diatoms (II), and amorphous material (III). Marker bar is 10 μm; distance scale 36 pixels = 10 μm. (**b**) 3D reconstructed image from (**a**); morphological film structure parameters: total surface coverage f = 18.3%, mean thickness = 43 μm, roughness fluctuation coefficient = 0.162, biovolume = 35,800 μm^3^, fractal dimension = 1.64, Hopkin’s aggregation index = 2.79, AC = 0.47, diatom abundance = 6.3 × 10^4^ ind. cm^−2^.

PHLIP analysis was used in this study to reveal the dynamics of three components of a phototrophic biofilm: bacteria, micro-algae and EPS (extracellular polymeric substances) [13]. In addition to the quantification of biological components, reflection images of biofilms visualize solid inorganic material (Figure 1). Different reflective materials were distinguished, including the bare glass substratum (I), silica frustules of diatoms present in the biofilm (II), and considerable amounts of amorphous material (III). Biovolume estimates showed that for the image stack in Figure 1, about 85% of the biofilm constituted of an inorganic reflective material, while an organic material part represented the remaining 15% of the total biovolume.

The fractal dimension value reflects a significantly complex and lengthy contact line of the island-like objects with the surrounding area, higher values point to a more developed border line geometry [10]. Hopkins’ aggregation index and AC data reveal the patchy nature of the biofilm matter spatial distribution since Hopkin’s index (>2) and AC (>0) [11,12]. Marine biofilms are communities of several species that adhere to surfaces and are held together within an extracellular polymeric substance (EPS) matrix.

**Figure 2:**
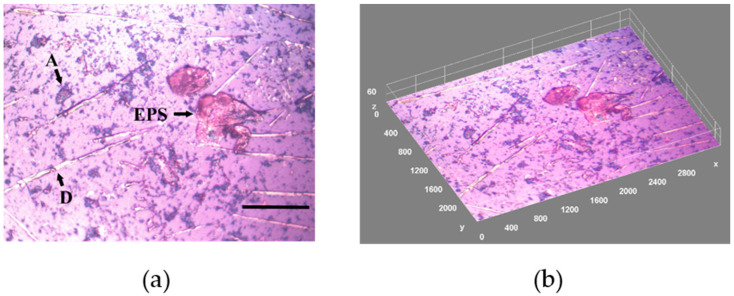
(**a**) A microscopic reflected image from the EPS stained with KMnO_4_ (brown areas) biofilm sample grown on glass slides (3-day old) in natural seawater (Baltic Sea, Gulf of Gdańsk, Poland) collected in August 2018. Black arrows point to the reflective biofilm structures: EPS comprising areas, silica frustules of diatoms (D), and amorphous inorganic material (A). Marker bar = 10 μm, distance scale 34 pixels = 10 μm. (**b**) 3D reconstructed image from (**a**). Morphological structure parameters: total surface coverage f = 4.8%, mean thickness = 14 μm, roughness fluctuation coefficient = 0.132, biovolume = 17,500 μm^3^, fractal dimension = 1.27, Hopkin’s aggregation index = 2.33, AC = 0.15, diatom abundance = 2.6 × 10^4^ ind. cm^−2^.

EPS components are difficult to visualize microscopically because of their low density and molecular complexity [14]. KMnO_4_ is a strong organic molecule oxidizing agent, and under neutral pH conditions, is known to react with EPS components yielding brown MnO_2_ precipitate deposition on the EPS. This oxidant is highly soluble in aqueous media but largely insoluble in non-polar solvents. To determine a fraction of EPS-glued biofilm species, we stained a 3-day old sample with the KMnO_4_ solution. The microscopic image of such a sample is shown in Figure 2a with the geometric parameters summarized in Figure 2b caption. The total film-covered area f = 11.4%, whereas for EPS f = 0.12% with a significant contribution from an amorphous inorganic material (f = 5.4%), and a number of diatoms (f = 1.8%).

The following trend of the marine biofilm structure evolution in the time elapsed, as expressed with the morphological parameters’ changes, was noticed: the-film forming matter covering increases (biovolume and f ↑), forming thicker (mean thickness ↑), and more rough the outermost surfaces (roughness coefficient η ↑), colonies of microbes form larger and separating from each other structures (both Hopkin’s index and AC coefficient ↑). It should be pointed out that no attempt was made to identify the microbes growing on the substrata (that was over the paper scope as well as the detailed seawater content analyses). However, a large variety of organisms were identified to compose the marine biofilm colony of different sizes and shapes, like bacteria (~1 μm), yeast (~3–5 μm), fungi (~12–18 μm), algae (~25 μm), ciliate (>200 μm) living apart from macroorganisms (barnacles or seaweeds) that is important in the light of surface roughness of substrata to be inhabited, as argued in [3]. Data on the marine classes and their occurrence in different seasons at the south Baltic region are provided in [3]. In the study area monocell algae were present composing microphytobenthos. Diatomophyceae and Dinophyceae dominated phytoplankton of the Baltic with a significant account of Cyanobacteria. The seasonality of organisms’ taxes are noticed: diatoms are found in spring, Cyanophyta in summer, and diatoms prevail in autumn.

### 3.2. Temporal Structure Evolution

**Figure 3:**
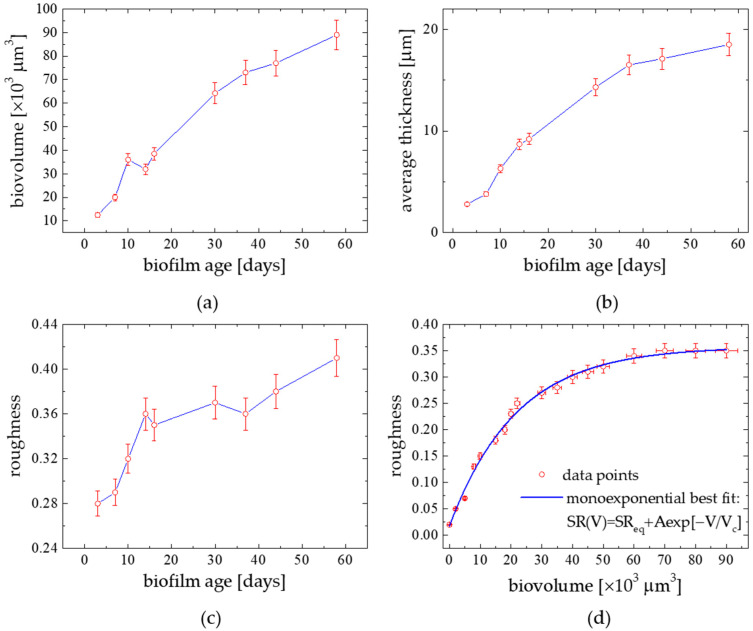
Structural parameters temporal development of a marine phototrophic biofilm grown on glass slides in natural seawater (Baltic Sea, Gulf of Gdańsk, Poland in August–October 2018); (**a**) biovolume, (**b**) average thickness, (**c**) R_rms_ roughness, and (**d**) correlation between biofilm roughness and biovolume; the line represents the monoexponential fit to the data (R = 0.99).

The quantitative description of a biofilm morphology can be obtained following the distribution of three principal components i.e., bacteria, microalgae and extracellular polymeric substances (EPS). The temporal development of a natural phototrophic biofilm is shown in Figure 3a–d. The phototrophic community succession was evidenced from small to larger species, both qualitatively from a visual inspection of images (as in Figure 1 and Figure 2) and quantitatively from an image analysis using PHLIP. The temporal development of a marine phototrophic biofilm was characterized by a continuous and linear increase tendency in biovolume (Figure 3a). Generally, the algal community evaluated from small to larger species which correlated to an increase in biovolume (R = 0.96) and demonstrated the biofilm thickness grow (R = 0.87) with time. EPS structures could be seen as bright spots. Biofilm thicknesses increased and levelled off with time. The biofilm thickness increased from around 3.4 μm, for a 3-day old biofilm, to 17 μm for the semi-steady biofilm case (40-day formed one). Bacteria were found to be a significant component only in the 4–old biofilm and located close to the substratum. On the other hand, EPS formed the thickest and outermost biofilm layers. Micro-algae generally inhabited the surface to intermediate layers of the biofilm. The results suggest that the contribution of bacteria in phototrophic biofilms may be low (1–4.9%) when compared to the algal and EPS components, which represented 20–76% and 19–77% of the biofilms, respectively. Growth of micro-algae dominated the development of biofilm. This led to a significant increase in biovolume, thickness and roughness of the biofilm. In Figure 3d there is a relation between roughness and biovolume averaged over the time of biofilm age formation constructed using more frequently sampled biofilm data set. The biofilm development leads to an increase in its heterogeneity since a strong correlation between roughness and biovolume was noticed (R = 0.99 for a monoexponential fit function: $SR\left(V\right)=SR_{eq}+Aexp\left[-V/V_{c}\right]$, with: $SR_{eq}$= 0.36 ± 0.01, A = −0.34 ± 0.01, $V_{c}$ = (21.8 ± 1.13)·10^3^ µm^3^). The observed small contribution of bacteria in mature biofilms compared to algae and EPS suggests a shift from a heterotrophic to an autotrophic system during the biofilm development. It agrees with the Baltic phytoplankton taxa seasonal changes [15]. Roughness high values indicated the importance of EPS for the structure of the biofilm pointing to an increase in the surface area allowing efficient attachment of micro-organisms to the substratum providing an additional source of nutrients [13]. Finally, these comprehensive studies confirmed that a strong correlation appeared between: biovolume vs. BWM (R = 0.78), coverage fraction f vs. BWM (R = 0.82), biofilm thickness vs. surface wettability parameters: surface film pressure П (R = 0.86), and surface energy γ_SV_ (R = −0.83), as evidenced in our previous work [3].

### 3.3. Seasonal Biofilm Morphology

**Figure 4:**
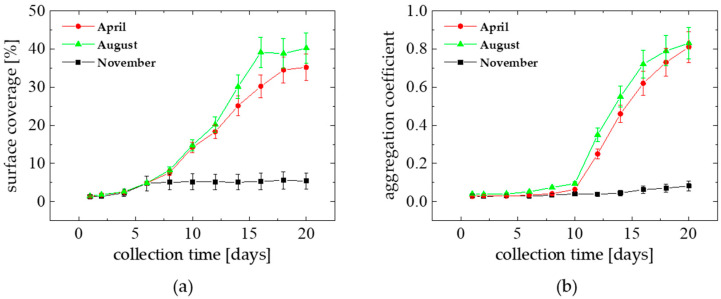
Biofilm surface coverage (**a**), and aggregation coefficient (**b**) versus sample collection time, for natural marine biofilm formation on a glass substratum in experiments performed in the Baltic Sea (Gulf of Gdańsk, Poland) in different organic matter production seasons (April–November 2018).

The long-term marine biofilm creation behavior is depicted in Figure 4a, for three measurement sessions conducted in April, August and November 2018, along 2–20 day-lasting periods. In April and August, the biofilm growth started to significantly increase after 5–6 days. In November, the exponential-like accumulation of the biofilm phase appeared to be substantially reduced. In particular, the final surface fractional coverage, registered after 16–18 days in April and August, reached quasi-constant values ranging around of 38–45%, respectively. In contrast, the surface coverage did not exceed a value of 5–6% in November. As found in the microscopic images, within the first 14 days, the biofilm structure consisted largely of bacteria ranging in a cell size from 0.7–2.4 μm. Later on (after 15–16 days), a significant increase in the biofilm species sizes was noticed on average over 12 μm.

It is supposed that the biofilm deposition efficiency, in particular biovolume, covering fraction, should be related to the trophic state indexes of the water body. The principal biogenic elements and *Chl. a* concentration with Primary Production (PP) data, averaged over each month of 2018, registered in the biofilm sampling site, are collected in Table 1. The most developed organic matter production was found in August. More comprehensive biofilm morphology data are required to establish a functional dependence between the structural parameters and the trophic state status indexes. However, as mentioned above, a significant correlation was already found between biovolume and biofilm wet mass (BWM) (R = 0.78), and coverage fraction f versus BWM (R = 0.82). Moreover, photoacoustic spectroscopy studies of biofilm-coated marine water-deployed substrata revealed a close coincidence between the photosynthetic system biofilm colonies (photosynthetic energy storage (ES), photoacoustic amplitude and phase spectra) and seasonal changes of primary production and biogenic elements concentrations in the studied Baltic Sea area [16,17].

In addition, a transition of the biofilm-forming species structure from dispersed spatially of low-dimension colonies to more compact aggregated structures can be learned from the AC coefficient dependence plotted versus the biofilm collection time shown in Figure 4b. For the semi-stable surface coverage state (observed after 14–16 days in Figure 4a), the continuous structural compaction of the film forming species into the more organized colonies was observed (AC ↑).

Various conditions may exist for different biofilm species distributions, as illustrated in Figure 1a–c of [12] for the exemplary structural configurations with the corresponding AC values. For a well-separated, uniformly spread microbe structure, AC = 0. Under certain conditions like light or nutrient limitation, the biofilm community tends to evolve to an aggregated form, AC = 0.55; afterwards, the microorganisms create an excessive extracellular matrix which leads to large cells clusters, where AC = 1. AC can sensitively describe the relative aggregation tendency of the biofilm colonies not dependent on the total biovolume. When the threshold of aggregate biovolume is fixed over 30 μm^3^ (as applied here), the AC sensitivity appears to be sufficient enough to distinguish the variability in aggregation patters before and after the sample treatments [12]. A dispersal agent, leading to the surface biomass reduction in the model strains, was capable of significantly decreasing the AC index [12]. AC could be successfully applied to quantify microbial aggregates formed under a wide range of conditions met in natural aquatic habitats [18,19] and wastewater treatment installations [20].

AC values revealed, in a quantitative way, the biofilm structure transition (related to quorum sensing, trophic state level, nutrient limitation, contamination stress, for instance) not evidenced in other film formation sensing techniques.

### 3.4. Substratum Wettability Effect

A detailed study on the selection of effective substrates for bioadhesion in aqueous media was already reported in [5]. Several abiotic (glass, metals, polymers, rubber) and biotic (wood, sediments, submerged algae surfaces) substrata were deployed for a standardized time (14 days) at the particular study place (Gulf of Gdańsk, Baltic Sea) in August 2018. First, the surface wettability energetics parameters of the model surfaces were evaluated, from the water contact angle (WCA) data, where the three measurable quantities were determined: θ_A_—the advancing contact angle (CA), θ_R_—receding CA, and γ_SL_—the probe liquid (water) surface tension, further processed with the contact angle hysteresis (CAH) approach [3]. Values of the considered surface wettability parameters: dynamic contact angles (θ_A_, θ_R_), contact angle hysteresis (CAH = θ_A_ − θ_R_), surface free energy (γ_SV_), 2D-adsorbed film pressure (Π), work of adhesion (W_A_), dispersive surface free energy component (γ_SV_^d^) are collected in Table 1 of [5].

In general, hydrophobic surfaces demonstrate a tendency to adsorb proteins and polymer materials from aqueous media, while those of hydrophilic nature gather more efficiently charged and polar materials.

The substratum surface energy effect on bioadhesion has been studied using various materials of widely varying γ_SV_ [21]. It was found that the test hydrophilic substrates (glass, metals, wood), exhibiting higher γ_SV_ (within the 40–58 mJ m^−2^ range) and both lower CA and γ_SV_^d^/γ_SV_ ratio revealed a high bioaccumulation capacity. For polymer substrates with γ_SV_ within the range 18–35 mJ m^−2^, a positive correlation (R = 0.66) was found between biomatter accumulation with γ_SV_, as argued in [22]. In addition, biofilm adhesion efficiency is not solely related to γ_SV_ but also to CAH [23]. Coating layers are most weakly bound to the surface if they are characterized by the lowest CAH values. An increase of CAH is accompanied with an increase in liquid to solid adhesive interaction strength (W_A_ ↑). Moreover, it was pointed to a link between bioadhesion and the substratum surface energy particular range [24]. A window in surface energy (between 20–30 mJ m^−2^) was noticed, where bioaccumulation reached a minimum. Substrata possessing γ_SV_ below 20 and above 30 mJ m^−2^ are said to display a substantial accumulation of biomass, with the maximum for surfaces with energies of approximately 60 mJ m^−2^ [24]. Recently performed studies on marine multispecies biofilm formation from natural seawater [25], with modified siloxane coatings samplers, revealed a significant effect of the polar component γ_SV_^p^ contribution to the total γ_SV_ of the substratum, i.e., γ_SV_^p^/γ_SV_ ↑ resulted in the lower bioaccumulation. For comparison, our model collecting surfaces had the following γ_SV_ (in mJ m^−2^) and γ_SV_^p^/γ_SV_ ratio values: metallic surfaces 33–39 (0.23–0.26); polymeric surfaces 18.9–25.5 (0.34–36); natural surfaces-Potamogeton lucens leaves 44–58 (0.32–0.14). The 2D surface pressure of the adsorbed film Π is related to the surface adsorption Γ (Gibbs excess; [26]): Γ ~ Π/R_G_T, where: R_G_ is the gas constant, and T—the absolute temperature. In our studies, BWM and biovolume were found positively correlated to Π. Natural submersed surfaces (soft bottom, rocks, sediments) revealed several times higher biofilm accumulation efficiency [27]. The similarity of biofilms on different substrata in seawater was observed for the longer stages of a colony formation (over 2 d), where a conditioning film of organic matter is quickly generated. This adsorbed molecular film masks the surface chemistry of the substratum [28]. As a result, colonizing organisms could perceive them as similar, and other factors as electricity, roughness, water chemistry, color etc. could mediate further colonization, as argued here.

Among the geometric-structural biofilm parameters, not all of them could be sensitive indicators of the substratum bioaccumulation. Our comprehensive study established that a strong positive correlation arose between: biovolume vs. biofilm wet mass (BWM) (R = 0.78), coverage fraction f vs. BWM (R = 0.82), biofilm thickness vs. П (R = 0.86), and biofilm thickness vs. γ_SV_ (R = −0.83). Generally, surfaces of metals and their alloys are coated with oxide films, which results in their hydrophilic properties with high surface energy. The final wettability depends on the history of the surface treatment when the surface processes induct adsorption, chemisorption, or chemical interaction of gases occurring in the ambient environment. Recently, several studies were performed on laser texturing of stainless steel, polymeric surfaces followed by surface modification with organic coating adapted to create composite surfaces with different surface roughness, morphologies, and wettability [29,30,31]. It is known that high energy surfaces (of hydrophilic character) are enriched in polar functional groups such as, –CO, –NH2, –OH, –COOH, whereas the hydrophobic surfaces contain non-polar groups (alkyl, fluoroalkyl, –SH, etc.). To conclude, assuming that the surface morphologies of all of the surfaces are similar, the surface hydrophilicity should increase with an increase of oxygen content, and an increased carbon content should lead to hydrophobic properties [6]. It should be noted that there is microbially influenced corrosion, or biocorrosion, leading to the accelerated deterioration of metals owing to the presence of biofilms on their surfaces. The detailed mechanisms of biocorrosion are still poorly understood but can affect the formed biofilm morphology [32,33].

### 3.5. Substratum Roughness Effect

**Figure 5:**
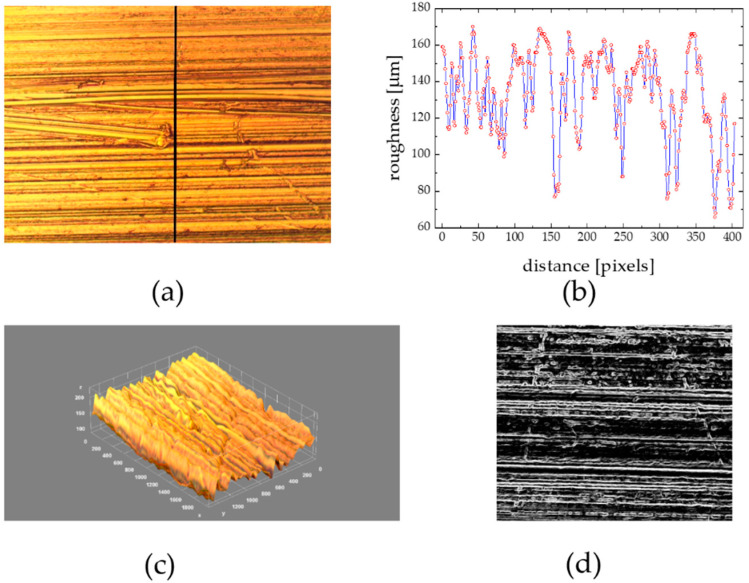
(**a**): A microscopic image of Al surface roughened with a sandpaper (grade 400), (**b**): roughness height profile along the line from (**a**), (**c**): 3D surface morphology from (**a**) derived with ImageJ routine, (**d**): (**a**) processed with “find edges” procedure; covered area x = 560 and y = 460 μm. Distance scale 180 pixels = 50 μm; R_rms_ roughness = 1.73 ± 0.43 μm; roughness fluctuation coefficient η = 0.178 ± 0.016.

Model biofilm deposition studies on rough abiotic materials were performed with Al metallic flat surfaces roughened (blasted) by hand undirectionally with sandpaper of grades 80–600. The microscale surface 3D architecture of such a surface of well-defined roughness (R_rms_) ranging from “fine” (0.22 μm) to “coarse” (19.6 μm) substrata with an irregularities surface profile plot along the line in a perpendicular direction to the direction of the paper scratching movement are depicted in Figure 5a,b.

The roughness factor R_f_ defined as the ratio of effective/projected areas (so-called geometric/nominal areas), for the exemplary surface, is equal to 2.73 and is >1, that is characteristic for a very rough surface [6]. Therefore, the apparent contact angle (and surface wettability) can be changed since air can be entrapped in capillary species between liquid and solid substratum. In such a case, the apparent CA can be expressed in the framework of the Cassie-Baxter model, as argued in our recent paper [6]. Analyzing the roughness profile from Figure 5b, it can be noticed that the mean capillary-like gaps had the mean cavity radius = 18.7 ± 0.2 μm. It is known that a large variety of organisms can form the marine biofilm colony differing in shape and size such as bacteria (1 μm), yeast (3–5 μm), fungi (12–18 μm), algae (~25 μm), cilitae (>200 μm) that is important in reference to the surface roughness of substrata to be inhabited [34]. The roughness profile revealed a series of valleys (5–8 μm deep) having valley floors and ridges ranging from 5–25 μm as well as of 6 μm diameter and 5–2.5 μm height pillars spaced with a distance (5–20 μm) from each other. The biofilm spores settled in valleys and against pillars, favorably on the pillar’s sides [35]. The effect of surface roughness on the wettability of hydrophilic substrata like Al should be addressed, as considered in [6]. These measurements performed, for R_rms_ from the range of 0.22–19.63 μm, revealed both WCA and γ_SV_ decrease and CAH ↑ as R_rms_ ↑, in agreement with data reported by others [36].

**Figure 6:**
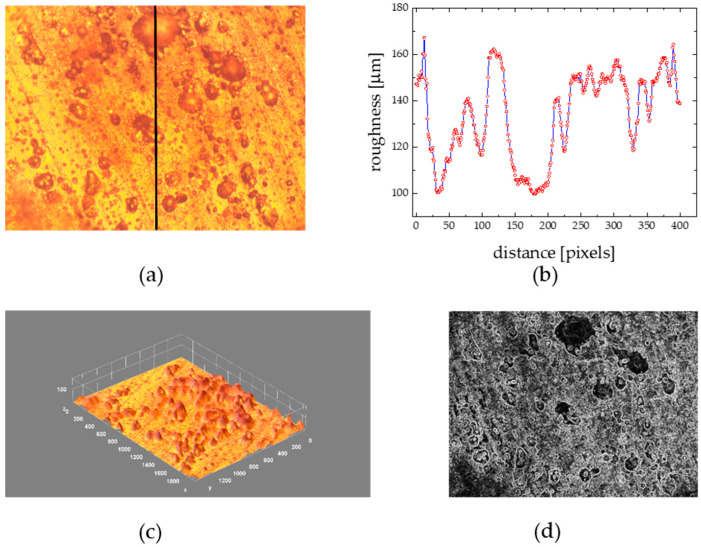
(**a**): A microscopic image of Al flat surface (reference) covered with a marine biofilm (14-day old), collected in Baltic sea water (Gulf of Gdańsk, Poland; August, 2018); (**b**): roughness height profile along the line from (**a**), (**c**): 3D surface morphology from (**a**) derived with ImageJ routine, (**d**): (**a**) processed with “find edges” procedure; covered area x = 660 and y = 520 μm. Distance scale 180 pixels = 50 μm; morphology parameters: surface coverage f = 28.7%, mean thickness = 9 μm, biovolume = 24,500 μm^3^, roughness fluctuation coefficient η = 0.141, fractal dimension = 1.16, Hopkin’s aggregation index = 2.14, AC = 0.25.

**Figure 7:**
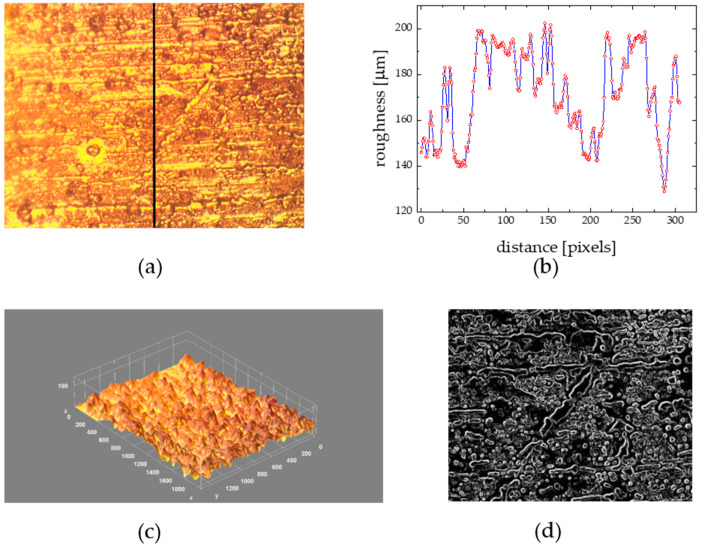
(**a**): A microscopic image of Al roughened surface covered with a marine biofilm (14-day old), collected in Baltic sea water (Gulf of Gdańsk, Poland; August, 2018); (**b**): roughness height profile along the line from (**a**), (**c**): 3D surface morphology from (**a**) derived with ImageJ routine, (**d**): (**a**) processed with ”find edges” procedure; covered area x = 670 and y = 530 μm. Distance scale 180 pixels = 50 μm; morphology parameters: surface coverage f = 64.6%, mean thickness = 16 μm, biovolume = 34,500 μm^3^, roughness fluctuation coefficient η = 0.132, fractal dimension = 1.26, Hopkin’s aggregation index = 2.34, AC = 0.13.

The substratum roughness effect on biofilm morphology was estimated on the microscopic images of the grooved Al palates and the flat one (as a reference), both depicted in Figure 6 and Figure 7, deployed simultaneously for the same collection period (14 days) in August 2018, at the same location.

The marine biofilm structure formed at the flat hydrophilic Al (WCA = 67.1, γ_SV_ = 45.6 mJ m^−2^ [6]) surfaces consisted of dispersed uniformly blob or drop-shaped structures of the diameter ranging from 10–89 μm. The border line between the biofilm matter and the substratum can be clearly distinguished from Figure 6d, where the function “find edges”, available in ImageJ program, was applied to the image from Figure 6a. The individual microorganisms are glued in an EPS matrix, where the “skin” tension effect leads to the colony volume/outermost surface ratio lowering to minimize the contact area of the structure to the substratum [21]. The diatoms were attached more strongly to hydrophobic surfaces [35], such as plastics studied in [5], than to hydrophilic substrata.

The structure of the biofilm collected at the grooved surface (Figure 7) was quite different. The collected material formed net-like structures composed of low-diameter (<10 μm) colonizers from unicellular organisms. The organic matter was homogeneously distributed over all the sample surfaces. The surface valleys or niches were completely filled in, and the initial regular roughened surface morphology (Figure 5c,d) could be no longer visible at the biofouled sample image (Figure 7c,d). The observed biofilm surface architecture evolution can be reflected in a quantitative way by the corresponding changes of the morphological parameters. In reference to the flat surface case, the surface is covered to a greater extent (f = 64.6 in comparison to 28.7%) with biomaterials forming thicker layers (thickness = 16 in reference to 9 μm), comprising higher biovolumes (biovolume = 34,500 ← 24,500 μm^3^), outermost surface flattening is observed (η = 0.132 ← 0.141), the border line between the aggregated biofilm structures and the surrounding environment is less complex (fractal dimension and Hopkin’s aggregation index ↓), and finally the spatial distribution of the biofilm colonies is more uniform (AC = 0.13 in reference to 0.25).

### 3.6. Substratum Color Effect

The effect of substratum color on the formation of microfouling communities was investigated using model plastic PVC (polyvinyl chloride) plates (R_rms_ ~ 0.2 μm) of different color ensuring very similar surface wettability properties. Values of γ_SV_, for all the substrates in spite of the particular color, took very similar values: γ_SV_ (blue) = 37.6, γ_SV_ (orange) = 40.6, γ_SV_ (red) = 39.4, γ_SV_ (brown) = 39.6, characteristic for polymeric and hydrophobic solids [5]. The studied surfaces bioaccumulation capacity is summarized in Table 2, where the surface coverage and BWM values, for the particular substratum color at the same sapling place and collection period, are given.

The highest and similar quantitatively biofilm accumulation was noticed for orange-red plastics, lower was exhibited by brown, and the lowest by blue ones. The substratum color effect studied with acrylic tiles revealed that the bacteria densities, and amount of Chlorophyll a, were collected more efficiently at the black surfaces than at white ones [37]. A deposited microorganism’s highest density was reported for the darker color (black, blue, red, grey) substrata whereas the lighter (yellow and white) ones were less populated [38]. It is supposed that the microorganisms containing pigments exhibit the chromatic adaptation mechanism to the colonized surface.

It can be noticed that the sampler color acts as a stressor for the biofilm colony organisms, which could respond with the particular more aggregated spatial colony structural organization (minimalizing the contact area with the surroundings), as established here for the blue and brown surfaces, where AC factor is almost twice higher than observed for the remaining ones. It means that the biofilm colony, using *cell-to-cell* communication (or *the quorum sensing*), can respond to the environmental parameters (nutrient or light limitation, temperature, pollution). As a result, AC could be a sensitive physical indicator of the biofilm intercellular quorum sensing effect.

The maximum light absorption peaks corresponding to the particular pigments occurring in marine biofilm colonies, accompanied by the characteristic colors, can be pointed to [39]: Chlorophyll a (430 and 660 nm; blue-green color), Chlorophyll b (455 and 640 nm; yellow-green), Phycocyanins (560 to 660 nm; blue-grey), Phycoerythrins (550 to 570 nm; red) and Carotenoids (430–570 nm; yellow-orange). Diatoms and cyanobacteria stand for the most abundant sessile species growing on submerged marine experimental substrata. There is evidence of the biofilm and diatom succession on polyethylene plastics in the sea likely to be present in these studies [40]. Cyanobacteria” or “blue-green algae” (Cyanophyceae) contain Chlorophyll a and phycocyanine. Diatoms contain two types of pigments involved in light harvesting and photoprotection: chlorophylls and carotenoids [41]. Benthic diatoms are a major component of biofilms that form on surfaces submerged in marine environments. In our study area, microphytobentos were composed from monocell algae. Dinophyceae and Diatomophyceae dominated phytoplankton of the Baltic with Cyanobacteria significant account. Organisms’ taxes exhibited following seasonality: diatoms are found in spring, Cyanophyta in summer, with diatoms prevailing in autumn [15].

**Figure 8:**
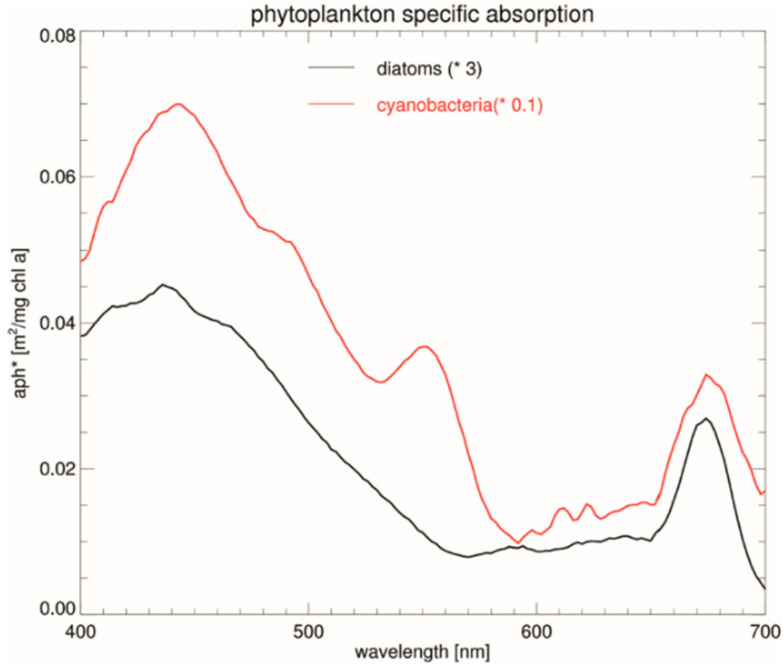
The spectra of pigment-specific phytoplankton absorption determined in marine water samples (reproduced after [42]). Symbols: cyanobacteria (red) and diatoms (black).

The exemplary diatom and cyanobacteria light absorption spectrum, obtained in remote sensing field experiments, is reproduced in Figure 8 after [42]. The presence of the particular pigments contained in the considered organisms was reflected in the corresponding spectral maxima, as stated above. In particular, a broad absorption band with the maxima, both for diatoms and cyanobacteria, were found at around λ = 440 nm, the local one at 550 nm, for cyanobacteria only, and the narrow one (675 nm) in the red-light region.

**Figure 9:**
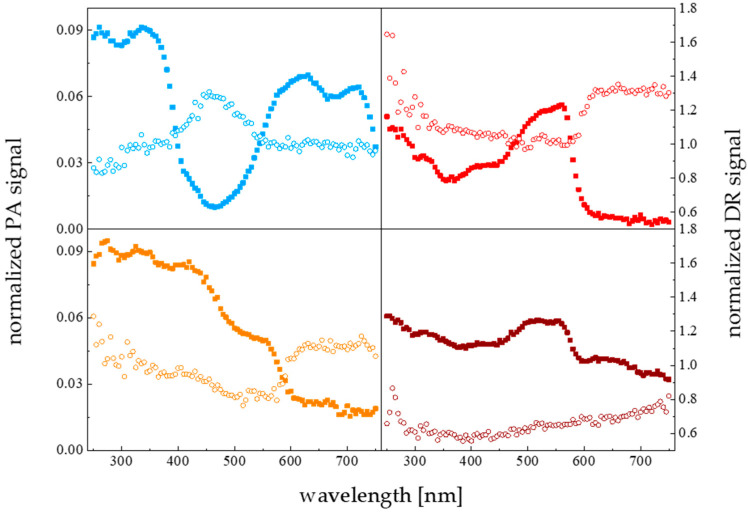
The photoacoustic spectra for solid PVC samples of different colors; sample color corresponds to symbol color. PAS data (circles) normalized with respect to carbon black, DR PAS data (filled squares) normalized to powdered quartz; modulation frequency 37 Hz.

The spectral light absorption features of the PVC colored deposition plates were determined with the photoacoustic spectroscopy (PAS) technique using a closed–type photoacoustic cell system, as described elsewhere [43]. The photoacoustic-based modalities allowed the spectral signatures of opaque materials to be determined. In addition, apart from a standard photoacoustic spectroscopy approach (PA), an alternative methodology based on the diffusive reflectance mode (DR) was used here to evaluate the spectral characteristics of the light beam reflected back from the solid sample [44]. The PAS absorption (squares) and PAS reflectance (circles) mode (DR) spectra, for PVC solid surfaces of different colors are shown in Figure 9.

The PAS adsorption spectra of the studied materials did not overlap significantly with the bands of the maximal pigment absorption intensity. On the contrary, the spectra of light reflected and scattered from the substratum of the particular color (compare orange and red relations) exhibited broad maxima centered at the wavelengths close to the characteristic ones for the organisms’ essential pigments (430 and 660 nm). Such a situation did not appear for the blue color sample, where the only maximum could be distinguished at ~470 nm. For the remaining brown surface, the reflected light spectrum intensity is low along the whole spectrum range with no distinguishable maxima. Considering the comprehensive biofilm morphology accumulation data, for the differentiated in color collecting surfaces, the colonizing organisms settled preferentially (higher f and BBM) at the available areas illuminated with the reflected-scattered light beam of a spectral composition fitting to the main pigments’ spectral absorption features. To summarize, the spectral signatures of the light reflected from the colonized surface in the biofouling phenomenon are responsible for the so-called chromatic adaptation mechanism of biofilm consortium to the substratum.

## 4. Conclusions

The spatial organization of complex natural biofilm colonies is critical to understanding the interaction of the individual taxa that comprise a community. Bacteria are micron-sized, and many of the forces and factors that underlie their distribution patterns operate at micron scales and are qualitatively different from the large scale factors, such as a trophic state status, primary production or man-made pollution stresses. 3D biofilm micron-scale morphology parameters derived by means of confocal microscopy assisted with image processing programs, allowed to specify biofilm growth mathematical models. A clear transition was observed from a heterotrophic community (enriched in bacteria) to an autotrophic community consisting largely of diatoms. It agrees with the Baltic phytoplankton taxa seasonal changes [15]. The biofilm structure transitions (related to quorum sensing, trophic state level, nutrient limitation, for instance), not revealed by other film formation sensing techniques, could be quantitatively evaluated. Moreover, the biofilm morphology parameters allowed the substratum roughness, surface wettability, chromatic organisms colony adaptation to substrata, cell-to-cell communication effects to be sensitively followed. These comprehensive studies confirmed that a strong correlation appeared between biovolume vs. biofilm wet mass (BWM) (R = 0.78), coverage fraction f vs. BWM (R = 0.82), biofilm thickness vs. surface wettability parameters: surface film pressure П (R = 0.86), and surface energy γ_SV_ (R = −0.83) in reference to our previous work [3]. A set of the biofilm morphological parameters could become a starting point to create a novel marine water biomonitoring technique based solely on physical attributes.

## Figures and Tables

**Table 1:** Baltic Sea trophic state parameters; mean (standard deviation); N, P, O biogenic elements (in mg m^−3^), *Chl. a* concentrations (mg m^−3^), PP—primary production (mg Cm^−2^ d^−1^); pH = 8.4, salinity = 6.2 PSU. Baltic Sea coastal waters (Gulf of Gdańsk, Poland; April–November 2018).

Month	N	P	O	*Chl. a*	PP
IV	228.7 (35.2)	97.7 (12.4)	13.1 (2.6)	6.0 (1.7)	751 (69.4)
VIII	39.3 (2.8)	62.4 (5.8)	11.3 (3.4)	4.1 (1.5)	972 (88.5)
XI	75.5 (6.9)	45.0 (3.9)	10.4 (1.9)	4.6 (2.0)	255 (30.1)

**Table 2:** The PVC substratum color effect on the biofilm collection efficiency (surface coverage f, biofilm wet mass (BWM), and AC). Baltic Sea studies (Gulf of Gdańsk, August 2018); collection time 14 days; mean (standard deviation).

Substratum	Surface Coverage [%]	BWM [mg cm^−2^]	AC
PVC blue	8.36 (2.19)	1.18 (0.24)	0.62 (0.07)
PVC orange	22.25 (2.87)	10.78 (2.21)	0.32 (0.05)
PVC red	20.46 (5.85)	13.69 (3.14)	0.29 (0.04)
PVC brown	16.84 (8.97)	6.85 (2.23)	0.53 (0.08)

## Data Availability

The data reported can be shared on demand after contacting the corresponding authors.
